# Pirfenidone inhibits TGF-β1-induced fibrosis via downregulation of Smad and ERK pathway in MDCK cells

**DOI:** 10.1007/s11259-024-10493-y

**Published:** 2024-08-12

**Authors:** Chae-Yoon Im, Se-Hoon Kim, Ki-Hoon Song, Min-Ok Ryu, Hwa-Young Youn, Kyoung-Won Seo

**Affiliations:** 1 Department of Veterinary Internal Medicine, College of Veterinary Medicine, Seoul National University, Seoul, Republic of Korea; 2 Research Institute, ViroCure Inc., Seoul, Republic of Korea

**Keywords:** Pirfenidone, Dog, Chronic kidney disease, Renal fibrosis, Epithelial-mesenchymal transition

## Abstract

The prevalence of chronic kidney disease (CKD) in dogs increases with age, and renal fibrosis is an important pathophysiological mechanism in this process. However, only a few drugs that can effectively inhibit fibrosis in the kidneys of dogs are currently available. In this study, we aimed to determine whether pirfenidone, a drug that has shown antifibrotic effects in various clinical studies, also exerts antifibrotic effects on canine renal tubular epithelial cells, Madin-Darby canine kidney cells (MDCK). To this end, we treated MDCK cells with various concentrations of pirfenidone, followed by transforming growth factor-beta1 (TGF-β1) to stimulate fibrotic conditions. A cell viability assay was performed to determine the effect of pirfenidone on cell survival. Fibrosis-related markers and TGF-β1 fibrotic pathway-related markers were assessed using qPCR, Western blot analysis and immunocytochemistry. A one-way analysis of variance (ANOVA) was performed, followed by Tukey’s post-hoc test for multiple comparisons. Pirfenidone treatment significantly reduced the expression of profibrotic markers such as α-smooth muscle actin, fibronectin, and collagen. Additionally, it upregulated the expression of E-cadherin, an epithelial marker. Furthermore, pirfenidone effectively inhibited the phosphorylation of key factors involved in the TGF-β1 signaling pathway, including Smad2/3 and ERK1/2. These results demonstrate that pirfenidone suppresses TGF-β1-induced fibrosis in MDCK cells by attenuating epithelial-mesenchymal transition and the relevant signaling pathways.

**Supplementary Information:**

The online version contains supplementary material available at 10.1007/s11259-024-10493-y.

## Introduction

Chronic kidney disease (CKD) in dogs is irreversible, with a progressive loss of kidney function and/or structure (Ettinger et al. 2016). CKD is the most recognized form of kidney disease in dogs (Ettinger et al. 2016) with a prevalence of approximately 0.02 to 1.44% and its incidence increases with age (O’Neill et al. 2013). Tubulointerstitial nephritis is the most common cause of CKD in dogs (Minkus et al. 1994). CKD progression frequently culminates in renal fibrosis, with tubulointerstitial fibrotic lesions observed on histopathological examination (Benali et al. 2014; Yhee et al. 2008, 2010).

Epithelial-mesenchymal transition (EMT) (Aresu et al. 2007) and extracellular matrix (ECM) accumulation (Yhee et al. 2010) occur during the progression of renal fibrosis. The injured renal epithelial cells turn into myofibroblasts with a mesenchymal phenotype (Benali et al. 2014). Simultaneously, increased fibroblasts and ECM deposition causes a progressive reduction of functional parenchyma (Benali et al. 2014).

The transforming growth factor beta-1 (TGF-β1) plays a pivotal role in the progression of kidney fibrosis in humans (Meng et al. 2016), and its involvement in fibrotic processes across multiple organs has been demonstrated in dogs (Krafft et al. 2014; Spee et al. 2006; Tang et al. 2023). This factor triggers fibrosis by activating the canonical Smad pathway and non-canonical pathways, such as the mitogen-activated protein kinase (MAPK) pathway (Meng et al. 2016). Modulation of the TGF-β1 pathway may be a potential target for impeding the progression of renal fibrosis.

Pirfenidone [5-Methyl-1-phenyl-2-(1 H)-pyridone] is an orally bioavailable drug with antifibrotic effects and has been approved by the Food and Drug Administration (FDA) for idiopathic pulmonary fibrosis in humans (US Food and Drug Administration 2017). In human patients with diabetic nephropathy, pirfenidone improves the estimated glomerular filtration rate (eGFR), presumably owing to its antifibrotic effects (Sharma et al. 2011). In addition, several animal studies have confirmed the ability of this drug to impede renal fibrosis by reducing the expression of EMT markers and ECM accumulation (Bi et al. 2022; Li et al. 2017; RamachandraRao et al. 2009; Shihab et al. 2002; Shimizu et al. 1998; Takakuta et al. 2010). Although its precise mechanism of action remains unclear, multiple studies have indicated that this drug targets several fibrotic pathways, including the Smad and MAPK pathways (Bi et al. 2022; Li et al. 2017).

Similarly, pirfenidone has been shown to exert antifibrotic effects in organs such as the heart and eyes (Hazra et al. 2021; Lee et al. 2006). However, to date, no studies have investigated the effects of pirfenidone on kidney fibrosis in dogs. Therefore, we aimed to evaluate the antifibrotic effect of pirfenidone in canine kidney epithelial cells and elucidate the underlying mechanism. Thus, we hope to explore the possibility of using pirfenidone as an antifibrotic drug for canine CKD in the future.

## Materials and methods

### Drugs and reagents

Pirfenidone was purchased from Tokyo Chemical Industry Co., Ltd. (Tokyo, Japan, #P1871) and dissolved in dimethyl sulfoxide (DMSO). The concentration of DMSO in the drug solution applied to the cells was 0.1% or less. TGF-β1 was purchased from Peprotech (Cranbury, NJ, USA, #100 − 21) and diluted according to the manufacturer’s instructions.

### Cell culture and treatment

The MDCK cell line was obtained from the Korean Cell Line Bank (Seoul, South Korea, #10034). Cells were cultured in Dulbecco’s modified Eagle’s medium (DMEM, Solbio, Suwon, South Korea, #DME-001) supplemented with 10% fetal bovine serum (FBS, Gibco, Thermo Fisher Scientific, Waltham, MA, USA, #16000-044), 100 U/mL penicillin-streptomycin (Gibco, #15140122). The cells were maintained in a humidified incubator at 37 °C with 5% CO_2_. For the experimental treatment, subconfluent cells were then cultured in FBS-free DMEM for 12 h. The cells were cultured until they reached approximately 40% confluency prior to drug treatment.

After that, cells were treated with medium alone, TGF-β1 1 ng/mL, or TGF-β1 1 ng/mL with 200–400 µg/mL of pirfenidone. Cells were pre-treated with pirfenidone for 1 h before the addition of TGF-β1. The medium was supplemented with 10% FBS in both the treatment and untreated normal groups during this phase. Specific treatment times are mentioned in the descriptions of each experimental technique.

### Cell viability assay

To determine a non-toxic dose of pirfenidone for subsequent experiments, we first conducted a cell viability assay. This assay was designed to establish which concentrations of pirfenidone did not affect cell viability, ensuring that subsequent experiments were performed using a safe and effective dose. The cells were seeded in 96-well plates for 12 h. Before treatment, cells were cultured in FBS-free DMEM for 12 h, after which the cells were treated with medium only, which contained DMSO 0.1%, pirfenidone 100, 200, 300, 400 and 500 µg/mL for 48 h. The medium used for drug treatment was supplemented with 10% FBS. First, Leica DMi1 microscope (Leica Microsystems, Wetzlar, Germany) was used to observe changes in cell visual morphology. Next, we performed a Cell Counting Kit-8 assay (CCK-8 assay, Dojindo Laboratories, Kumamoto, Japan, #CK04) which utilizes water-soluble tetrazolium salt-8 (WST-8) to quantitatively confirm cell viability. Before the CCK-8 assay, the medium was replaced with pirfenidone-free DMEM. We assessed the cell viability through measuring absorbance at 450 nm after 2 h of WST-8 treatment. The absorbance of the medium alone was subtracted from each group’s absorbance, and the viability of the control group was set to 100%. Viability was then compared across the different drug treatment groups.

### Quantitative real-time PCR (RT-qPCR)

Cells expressing various markers were treated for 24 h as mentioned above, except for fibronectin, which was treated for 6 h. After treatment, total RNA was extracted using the AccuPrep Universal RNA Extraction Kit (Bioneer, Daejeon, Korea, #K-3141) and reverse-transcribed to cDNA using the Compact cDNA Synthesis kit (Smartgene, Daejeon, Korea, #SG-cDNAC100) following the manufacturer’s protocol. Quantitative PCR was performed on a Qunatstudio1 (Applied Biosystems, Foster City, CA, USA, #A40427) using SYBR Green qPCR Master Mix (Smartgene, #SG-SYBR-ROXH). The amplification conditions were set to an initial denaturation at 95 °C for 2 min, followed by 40 cycles of denaturation at 95 °C for 5 s and annealing/extension at 60 °C for 20 s. Specific primers for the target genes and GAPDH as an internal control were used (Table S1). The expression levels were normalized to GAPDH and calculated using the 2^−ΔΔCt^ method. Melting curve analysis was conducted to ensure the specificity of the PCR products. Data are presented as fold-change relative to the control. All samples were analyzed in triplicates.

### Western blotting

The cells were treated for 48 h as described above and were lysed using a cell lysis buffer (Smartgene, #SG-PR-CELI). The lysates were incubated on ice for 30 min and then centrifuged at 12,000 RPM (15,928 × *g*) at 4 °C for 20 min. Supernatants were collected, and protein concentrations were determined using a Pierce BCA Protein Assay Kits (Pierce Biotechnology, Thermo Fisher Scientific, Waltham, MA, USA, #23227).

Equal amounts of proteins (6–20 µg) were separated by SDS-PAGE on 6–12% polyacrylamide gels and transferred to Immobilon^®^-P PVDF Membrane (MilliporeSigma, Darmstadt, Germany, #IPVH00010). The membranes were blocked with 5% skim milk (LPS solution, Daejeon, Korea, #SKI500) or bovine serum albumin (BSA, Georgiachem, #G.C-BS1005) in TBST for 1 h at room temperature. Membranes were incubated overnight at 4 °C with primary antibodies, such as anti-fibronectin (1:200 dilution, Santa Cruz Biotechnology, Inc., Santa Cruz, CA, USA, #sc-59826), anti-collagen3 (1:100 dilution, Santa Cruz Biotechnology, Inc., #sc-271249), anti-α-SMA (1:800 dilution, ABclonal Technology, Wuhan, China, #A7248), anti-E-cadherin (1:200 dilution, Santa Cruz Biotechnology, Inc., #sc-59778), anti-vimentin (1:100 dilution, Santa Cruz Biotechnology, Inc., #sc-373717), anti-Smad2/3 (1:800 dilution, ABclonal Technology, #A7536), anti-phospho-Smad2/3 (1:800 dilution, ABclonal Technology, #AP1343), anti-ERK1/2 (1:1,000 dilution, Cell Signaling Technology, Danvers, MA, USA, #4695), anti-phospho-ERK1/2 (1:1,000 dilution, Cell Signaling Technology, #9101) and anti-GAPDH (1:5,000 dilution, ABclonal Technology, AC002). For those antibodies not specified to target canine proteins, we conducted validation experiments in MDCK cells. After washing with TBST, the membranes were incubated for 1 h at room temperature with secondary antibodies, including horseradish peroxidase-conjugated anti-mouse antibody (1:5,000 dilution, ABclonal Technology, #AS003), HRP-conjugated anti-rabbit antibody (1:5,000 dilution, ABclonal Technology, #AS014) and HRP conjugated anti-rat antibody (1:10,000, Invitrogen, Thermo Fisher Scientific, Waltham, MA, USA, #31470). Protein bands were visualized using ECL High Femto Solution (Smartgene, #SG-PR-HECL) and imaged using a Las 4000 imager (GE Healthcare Biosciences, Milwaukee, WI, USA). Band intensities were quantified using ImageJ software, and the relative protein levels were normalized to those of GAPDH.

### Immunofluorescence assay

Cells were seeded on coverslips in 35 mm dishes at an optimal density for growth. After treatment, the cells were washed with PBS and fixed with 4% paraformaldehyde for 10 min at room temperature. The cells were then permeabilized with 0.1% Triton X-100 for 10 min. The cells were blocked with 1% BSA and 300 mM glycine in PBS for 30 min to prevent nonspecific binding. The primary antibody anti-fibronectin (1:50 dilution, Santa Cruz Biotechnology, Inc., #sc-59826) was applied and incubated for 1 h at room temperature. After washing three times with PBS, the cells were incubated with FITC anti-mouse secondary antibodies (1:100 dilution, ABclonal Technology, #AS001) for 1 h at room temperature. Validation for both primary and secondary antibodies was performed in the immunofluorescence (IF) experiments. Cells were washed three times with PBS, and coverslips were mounted with a mounting solution containing DAPI (Invitrogen, #00-4959-52). Fluorescence was visualized using an EVOS FL (EVOS, Thermo Fisher Scientific, Waltham, MA, USA).

### Statistical analysis

Data are presented as mean ± standard error of the mean (SEM). A one-way analysis of variance (ANOVA) was performed, followed by Tukey’s post-hoc test for multiple comparisons. All statistical analyses were performed using GraphPad Prism (version 10.1.2, GraphPad Software Inc., San Diego, CA, USA). Statistical significance was set at *P* ≤ 0.05.

## Results

### Effects of pirfenidone on cell viability

**Fig. 1:**
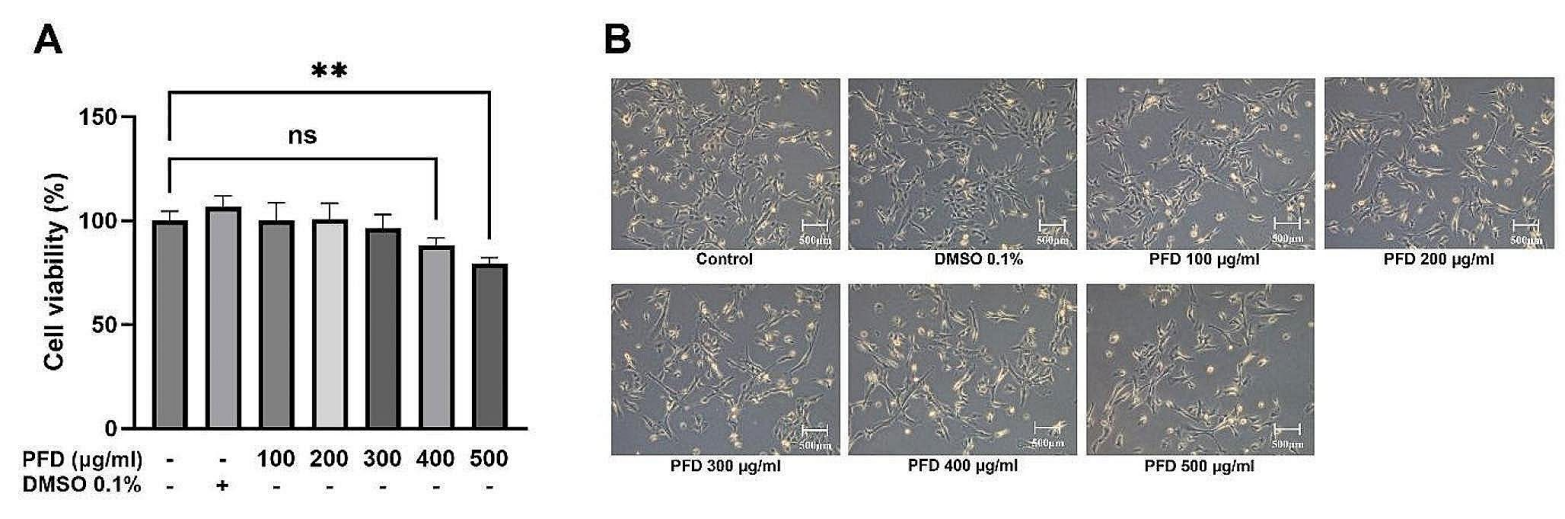
Effects of pirfenidone (PFD) on cell viability and appearance in MDCK cells. Cells are treated with medium only, 0.1% DMSO (vehicle), or PFD (100, 200, 300, 400–500 µg/ml) for 48 h. **A** Cell viability is measured via a CCK-8 assay. Data are presented as mean ± SEM. The experiments are performed three times, with similar results. **P < 0.01. **B** Evaluation of cell morphology using phase contrast microscopy. Scale bar: 500 μm

To investigate the effect of pirfenidone on the viability of MDCK cells, the cells were treated with various concentrations of pirfenidone for 48 h. At a pirfenidone concentration of 500 µg/mL, significant inhibition of cell viability was seen compared to the control, while no considerable inhibition was observed at lower concentrations, up to a pirfenidone concentration of 400 µg/mL (Fig. 1A). Observation by phase contrast microscopy revealed no differences in cell morphology, such as changes in shape, compared to the control up to a concentration of 400 µg/mL (Fig. 1B). Therefore, subsequent experiments were conducted with drug concentrations of 200 and 400 µg/mL.

### Pirfenidone inhibits TGF-β1-induced gene expression of EMT markers and ECM components

**Fig. 2:**
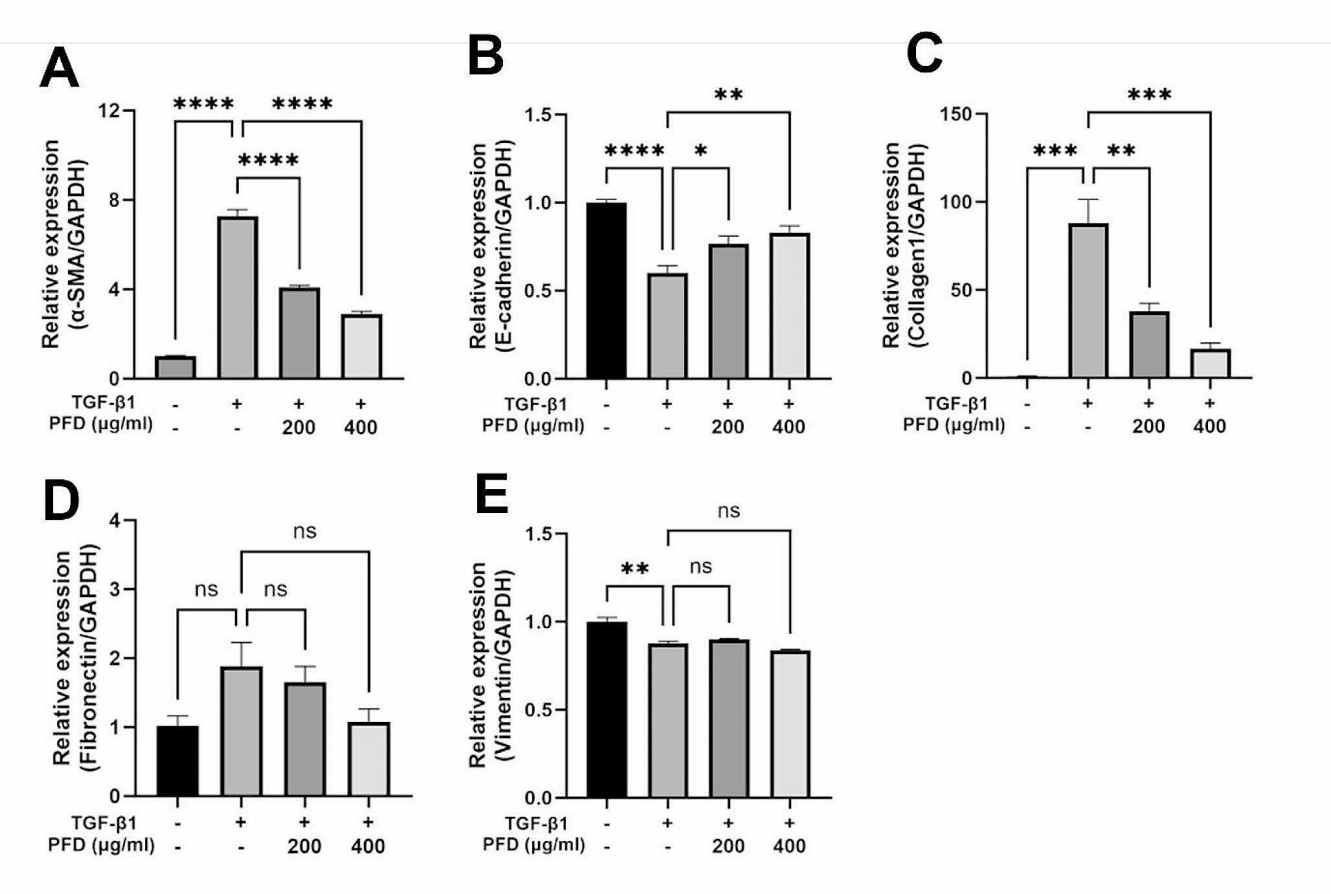
Effects of pirfenidone (PFD) on the TGF-β1 induced mRNA expression of EMT markers and ECM components tested using qPCR. Each graph represents relative mRNA expression of α-SMA (**A**), E-cadherin (**B**), Collagen1 (**C**), Fibronectin (**D**), and Vimentin (**E**). Cells are treated with medium only, TGF-β1 1 ng/mL, and TGF-β1 1 ng/mL with PFD 200–400 µg/mL. PFD is added 1 h before the TGF-β1 stimulation and the total incubation period was 24 h except for fibronectin (6 h). Data are presented as mean ± SEM. The experiments were performed three times, with similar results. ****P < 0.0001, ***P < 0.001, **P < 0.01 and *P < 0.05. ECM, extracellular matrix; EMT, epithelial-mesenchymal transition

The effect of TGF-β1 stimulation on the mRNA expression of fibrosis-associated EMT markers and ECM components in MDCK cells and the impact of pirfenidone treatment on this mRNA expression were assessed. Regarding the EMT markers, TGF-β1 significantly upregulated the mRNA expression of α-smooth muscle actin (α-SMA) (Fig. 2A) and downregulated E-cadherin (Fig. 2B). These trends were reversed in a dose-dependent manner by pirfenidone treatment. In the case of collagen1, an ECM component, its mRNA expression was increased by TGF-β1 and was dose-dependently inhibited by pirfenidone (Fig. 2C). In contrast, fibronectin did not show a notable increase in expression in response to TGF-β1 stimulation or a decrease in expression after pirfenidone treatment for 6 h (Fig. 2D). After 12, 24, and 48 h of treatment, the fibronectin mRNA expression was increased in response to TGF-β1 stimulation but it did not show a consistent tendency following pirfenidone treatment (Fig. S1A-C). Vimentin expression was decreased by TGF-β1 stimulation and did not show any difference after pirfenidone treatment (Fig. 2E). These results demonstrate that changes in the expression of ECM components and EMT markers induced by TGF-β1 can be regulated at the transcription level by pirfenidone.

### Pirfenidone inhibits TGF-β1-induced protein expression of EMT markers and ECM components

**Fig. 3:**
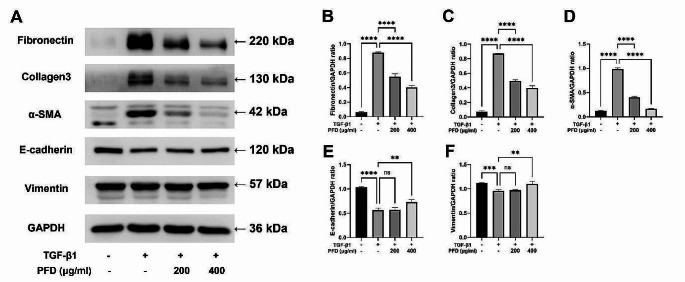
Effect of pirfenidone (PFD) on the TGF-β1 induced protein expression of EMT markers and ECM components tested using Western blot. Cells are treated with medium only, TGF-β1 1 ng/mL, and TGF-β1 1 ng/mL with PFD 200–400 µg/mL. PFD is added 1 h before the TGF-β1 stimulation, and the total incubation period was 48 h. **A** Protein levels of EMT markers and ECM. The experiments are performed three times, with similar results. Quantitative analysis of protein expression is performed, and the results of inter-group comparisons are shown. Each represents a relative ratio to GAPDH of fibronectin (**B**), Collagen3 (**C**), α-SMA (**D**), E-cadherin (**E**), and Vimentin (**F**). Data are presented as mean ± SEM. ****P < 0.0001, ***P < 0.001, **P < 0.01. ECM, extracellular matrix; EMT, epithelial-mesenchymal transition

Following the gene expression analysis, Western blotting was performed to determine the effect of pirfenidone on the expression of EMT markers and ECM components at the protein level (Fig. 3A). TGF-β1 treatment significantly increased the expression of ECM components, including fibronectin (Fig. 3B) and collagen3 (Fig. 3C). Pirfenidone effectively inhibited this upregulation in a dose-dependent manner. Furthermore, regarding EMT markers, activation of TGF-β1 signaling led to an increase in α-SMA expression (Fig. 3D) and decrease in E-cadherin expression (Fig. 3E). These trends were reversed in a dose-dependent manner after the pirfenidone administration. There was little change in the E-cadherin expression at lower pirfenidone concentrations; however, a significant change was observed at higher concentrations. In contrast, vimentin expression was decreased by TGF-β1 stimulation and increased at higher pirfenidone concentration (Fig. 3F).

### Pirfenidone inhibits TGF-β1-induced expression of EMT markers and ECM components in immunocytochemistry

**Fig. 4:**
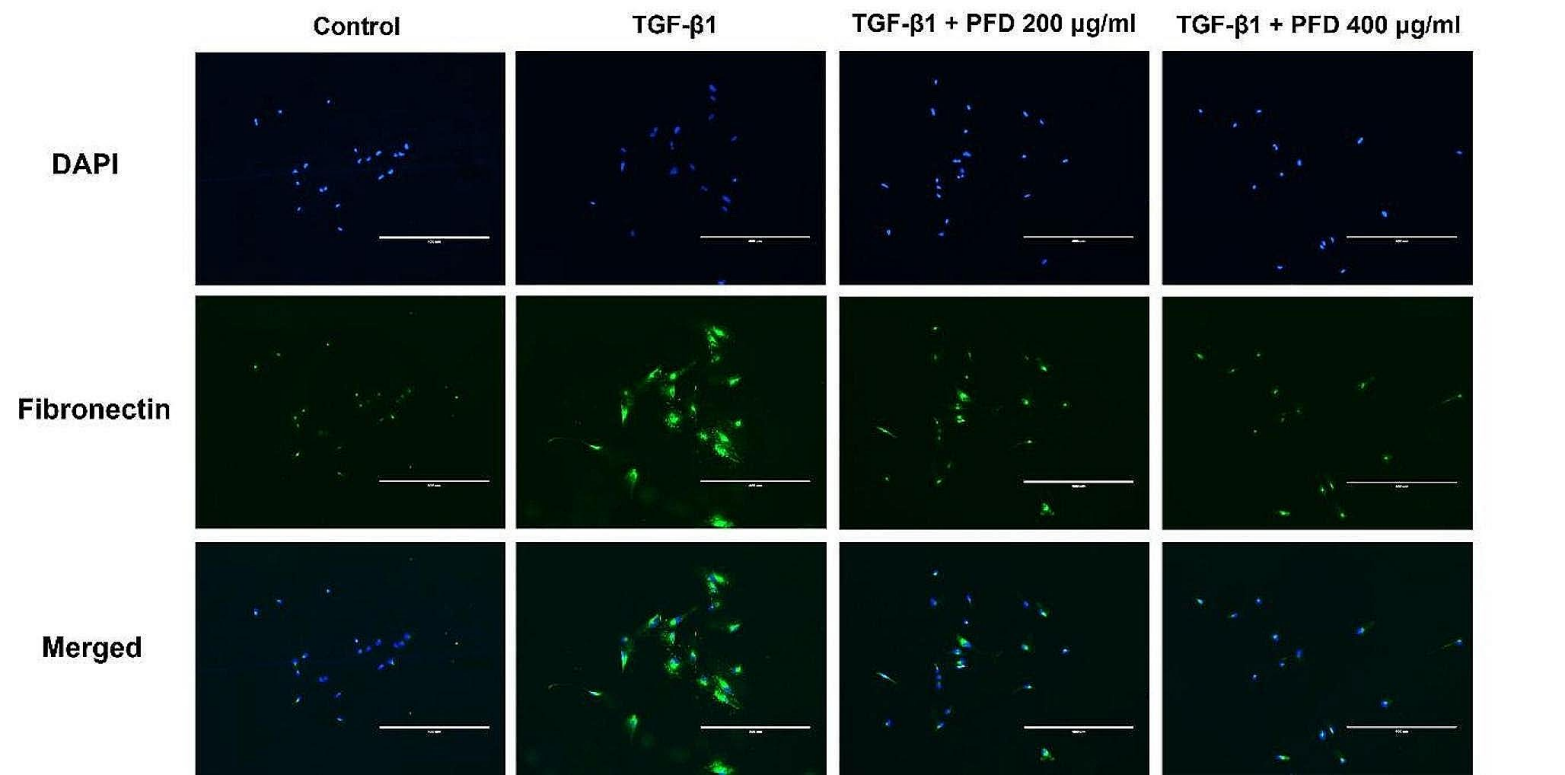
Effect of pirfenidone (PFD) on the TGF-β1 induced fibronectin expression on immunocytochemistry. Cells are treated with medium only, TGF-β1 1 ng/mL, and TGF-β1 1 ng/mL with PFD 200–400 µg/mL. PFD is added 1 h before the TGF-β1 stimulation and the total incubation period was 48 h. Then, cells are immunostained with an anti-fibronectin antibody and nuclei are counterstained with DAPI. The experiments are performed three times, with similar results. Scale bar: 400 μm

One of the key features of renal fibrosis induced by TGF-β1 is the increased synthesis of major markers of fibrotic ECM, such as fibronectin, within the cells. Therefore, we investigated the intracellular expression changes of fibronectin using immunocytochemistry (Fig. 4). Fluorescence signaling indicated that fibronectin expression was increased after TGF-β1 treatment, and notably, this expression was reduced following pirfenidone treatment. These results demonstrated that pirfenidone effectively inhibits TGF-β1 induced fibrosis.

### Smad and ERK signaling pathways are involved in the antifibrotic mechanisms of pirfenidone

**Fig. 5:**
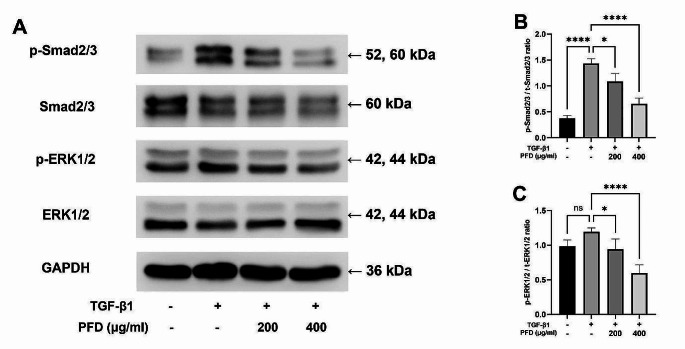
Effect of pirfenidone (PFD) on the TGF-β1 induced protein expression of TGF-β1 signaling pathway components tested using Western blot. Cells are treated with medium only, TGF-β1 1 ng/mL, and TGF-β1 1 ng/mL with PFD 200–400 µg/mL. PFD is added 1 h before the TGF-β1 stimulation and the total incubation period is 48 h. **A** Protein levels of TGF-β1 signaling pathway markers. The experiments are performed three times, with similar results. Quantitative analysis of protein expression is performed, and the results of inter-group comparisons are shown. Each represents the relative ratio of p-Smad/t-Smad (**B**) and p-ERK/t-ERK (**C**). Data are presented as mean ± SEM. ****P < 0.0001, *P < 0.05

To elucidate the antifibrotic mechanism by which pirfenidone exerts its antifibrotic effects, we investigated the changes in the downstream signaling pathways of TGF-β1 (Fig. 5A). To determine the involvement of the Smad pathway, a TGF-β1 canonical fibrosis pathway, we examined the expression levels of phospho-Smad2/3. When quantified in total form, phosphorylation of Smad2/3 was increased in response to TGF-β1 treatment and decreased in a dose-dependent manner upon pirfenidone treatment (Fig. 5B). This suggested that the Smad pathway plays a key role in the process by which pirfenidone inhibits TGF-β1-induced fibrosis. Next, to determine the involvement of the non-canonical MAPK pathway, we examined the expression level of phosphor-ERK1/2, another downstream mediator of TGF-β1 signaling pathway. Upon quantification of the total form, we observed an increase in phosphorylated-ERK1 following TGF-β1 treatment, which subsequently decreased in a dose-dependent manner following treatment with pirfenidone (Fig. 5C). These results suggested that ERK1/2, in addition to the Smad pathway, is involved in the antifibrotic action of pirfenidone.

## Discussions

In canine CKD, fibrosis is a crucial pathophysiological mechanism contributing to the end-stage disease’s progression. However, few studies have identified drugs with antifibrotic effects in canine kidneys. In this study, the antifibrotic effects of pirfenidone were assessed in MDCK cells. EMT markers and ECM accumulation analysis confirmed that pirfenidone exerted its antifibrotic effects through the Smad and ERK pathways.

In fibrotic kidneys, EMT plays a key role in the transformation of damaged renal epithelial cells into myofibroblasts (Aresu et al. 2007; Benali et al. 2014). α-SMA expression was significantly upregulated, and that of E-cadherin downregulated by TGF-β1 in MDCK cells, indicating the induction of EMT. Pirfenidone inhibited this process, suggesting a suppressive effect on EMT. In rat models of experimentally induced renal fibrosis, such as through unilateral ureteral obstruction (UUO) (Bi et al. 2022; Li et al. 2017) or partial nephrectomy (Chen et al. 2013), pirfenidone demonstrated similar therapeutic effects. These effects were evidenced by the reduced expression of EMT markers, including α-SMA, E-cadherin, S100A4 and tight junction protein1 (TJP1). Furthermore, similar observations were confirmed in human renal tubular HK-2 cell (Li et al. 2017). In a rat model of renal fibrosis induced by renal allograft transplantation, the antifibrotic effect of pirfenidone was demonstrated by a decrease in vimentin expression after pirfenidone administration (Qiu et al. 2019). However, this study found no change in vimentin expression following TGF-β1 or pirfenidone treatment in canine cells, suggesting potential species-specific differences in marker responses.

Renal interstitial fibrosis, which is characterized by the accumulation of collagen and other ECM components, can be triggered by an imbalance in ECM degradation and production (Martins et al. 2013). In our study, we observed that TGF-β1 induced a significant increase in ECM factors such as collagen and fibronectin, which were effectively reversed by pirfenidone treatment. Similar effects have been observed in various rat models where fibrotic states are induced through partial nephrectomy (Chen et al. 2013; Takakuta et al. 2010), drug administration (Brook et al. 2005; Shihab et al. 2002), UUO (Bi et al. 2022; Li et al. 2017; Shimizu et al. 1998), dietary changes (Ji et al. 2013), renal allograft transplantation (Qiu et al. 2019) and immune responses (Leh et al. 2005). In these previous studies, pirfenidone treatment was shown to reduce renal fibrosis, as confirmed by ECM-related markers such as collagen, plasminogen activator inhibitor-1 (PAI-1), biglycan, matrix metalloproteinase (MMP)/tissue inhibitor of metalloproteinase (TIMP), and connective tissue growth factor (CTGF). These antifibrotic effects were also confirmed in experiments with human kidney tubular cells (HK-2) (Li et al. 2017) and rat kidney tubular cells (NRK52E) (Takakura et al. 2012), utilizing similar markers. In these studies, collagen showed the most significant decrease after pirfenidone treatment. Consistent with these findings, our research on canine cells supports this effect, demonstrating a comparable reduction in fibrosis.

The progression of kidney fibrosis is significantly driven by TGF-β1, with the Smad pathway identified as the primary mechanism through which TGF-β1 induces fibrosis (Lan and Chung 2012). TGF-β1 stimulates the phosphorylation of Smad2 and Smad3, which then form a complex with Smad4, regulating gene expression for fibrotic proteins (Meng et al. 2016). Conversely, Smad7 acts as an inhibitory Smad, downregulating the pathway by preventing the phosphorylation and activation of Smad2 and Smad3 (Meng et al. 2016). In our study, we observed that TGF-β1 upregulated the ratio of phosphorylated Smad2/3 to total Smad2/3, an effect markedly inhibited by pirfenidone. These findings suggest that pirfenidone exerts its antifibrotic effect predominantly by modulating the Smad pathway. Similarly, a rat model of renal fibrosis induced by a high-salt diet showed a decrease in phosphorylated Smad2 and Smad3 following pirfenidone treatment, indicating that pirfenidone can mitigate fibrosis by altering the Smad signaling pathway (Ji et al. 2013). Furthermore, a study using UUO rat models revealed that pirfenidone enhanced the expression of Smad7, further supporting its role in regulating the Smad pathway (Bi et al. 2022).

Additionally, TGF-β1 activates the MAPK pathway, including ERK, JNK and p38, further contributing to renal fibrosis (Meng et al. 2016). In this study, the ratio of phosphorylated ERK1/2 to total ERK1/2 was increased by TGF-β1 and suppressed by pirfenidone treatment, suggesting that the ERK pathway was involved in the antifibrotic mechanism of pirfenidone. In a study using a UUO rat model and HK-2 cells, pirfenidone exhibited an antifibrotic effect by suppressing the MAPK signaling pathway, specifically targeting ERK, JNK, p38 and c-Jun (Li et al. 2017). Another study using the NRK52E cell line showed that pirfenidone altered the MAPK signaling pathway by modulating ERK and c-Fos (Takakura et al. 2012).

To support the relevance of MDCK cells as a model for studying fibrosis in canine CKD, we reviewed the expression levels of fibrotic markers in ex-vivo canine tubular epithelial cells. Studies show that vimentin and α-SMA are absent in healthy kidneys but present in degenerate and inflamed tubules, with α-SMA found in peritubular cells in fibrotic regions (Aresu et al. 2007). Additionally, ESRD cases exhibit extensive fibrosis, including destruction of proximal tubules, extracellular matrix deposition, and immune cell infiltration (Yhee et al. 2010). These findings align with the responses observed in our MDCK cell model, supporting its validity for fibrosis research in dogs.

Several studies have validated the safety of pirfenidone in dogs in vivo. In FDA safety testing of pirfenidone, no observed adverse effect level (NOAEL) for oral pirfenidone administered to dogs over 9 months was established at 200 mg/kg/day, yielding a maximum blood concentration (C_max_) of 156.3 to 176.2 µg/mL on the final day of treatment (US Food and Drug Administration 2014). Considering that the highest concentration of pirfenidone used in previous study was 200 mg/kg/day, further experiments at higher concentrations are necessary to accurately determine the NOAEL of pirfenidone in dogs. Moreover, additional studies in cell culture involving pirfenidone concentrations below 200 µg/mL, the lowest concentration used in our study, could help assess its efficacy at lower doses. Another study confirmed that the oral administration of pirfenidone at dosages ranging from 75 to 96 mg/kg/day for three weeks resulted in no observable drug-related adverse effects (Lee et al. 2006). Similarly, 0.5% pirfenidone eye drops administered thrice daily for two weeks showed no adverse effects (Hazra et al. 2021). Based on these findings, we anticipate that after further toxicity study concentrations, pirfenidone could be considered for use in dogs as a drug with antifibrotic effects.

Our study had several limitations. First, our in vitro experiments using MDCK cells may not have fully replicated the complex in vivo environment of canine CKD. Second, although our focus was on the Smad and ERK pathways, other pathways and interactions may play a role in the antifibrotic effects of pirfenidone. Future studies should consider these additional pathways to provide a more comprehensive understanding of the mechanism of action of pirfenidone.

## Conclusion

In conclusion, our study demonstrates that pirfenidone exhibits significant antifibrotic activity in MDCK cells, which is primarily achieved through the inhibition of the EMT process and reduction of ECM accumulation. The Smad pathway has been identified as a critical contributor to the antifibrotic action of pirfenidone, with the ERK1/2 pathway also being recognized as playing a significant role. These findings highlight the potential of pirfenidone as a therapeutic agent for treating renal fibrosis in dogs with end-stage CKD.

## Supplementary Information

Table. S1

Fig. S1

## Data Availability

No datasets were generated or analysed during the current study.

## References

[CR1] Aresu L, Rastaldi MP, Scanziani E, Baily J, Radaelli E, Pregel P, Valenza F (2007) Epithelial–mesenchymal transition (EMT) of renal tubular cells in canine glomerulonephritis. Virchows Arch 451(5):937–942. 10.1007/s00428-007-0482-8 17701211 10.1007/s00428-007-0482-8

[CR2] Benali SL, Lees GE, Castagnaro M, Aresu L (2014) Epithelial mesenchymal transition in the progression of renal disease in dogs. Histol Histopathol 29(11):1409–1414. 10.14670/HH-29.1409 24872206 10.14670/HH-29.1409

[CR3] Bi L, Huang Y, Li J, Yang X, Hou G, Zhai P, Zhang Q, Alhaji AA, Yang Y, Liu B (2022) Pirfenidone attenuates renal tubulointerstitial fibrosis through inhibiting miR-21. Nephron 146(1):110–120. 10.1159/000519495 34724669 10.1159/000519495

[CR4] Brook NR, Waller JR, Bicknell GR, Nicholson ML (2005) The experimental agent pirfenidone reduces pro-fibrotic gene expression in a model of tacrolimus-induced nephrotoxicity. J Surg Res 125(2):137–143. 10.1016/j.jss.2004.12.007 15854665 10.1016/j.jss.2004.12.007

[CR5] Chen J-F, Ni H-F, Pan M-M, Liu H, Xu M, Zhang M-H, Liu B-C (2013) Pirfenidone inhibits macrophage infiltration in 5/6 nephrectomized rats. Am J Physiol Ren Physiol 304(6):F676–F685. 10.1152/ajprenal.00507.2012 10.1152/ajprenal.00507.2012 23152296

[CR6] Ettinger SJ, Feldman EC, Côté E (2016) Textbook of veterinary internal medicine. Elsevier Health Sciences, Amsterdam, pp 4693–4474

[CR7] Hazra S, Maity N, Konar A (2021) Topical pirfenidone drops diminish opacity in dogs with corneal defects, in vitro safety, and in vivo efficacy– a pilot study. Rev Veterinaire Clin 56(2):63–68. 10.1016/j.anicom.2020.12.001

[CR8] Ji X, Naito Y, Weng H, Ma X, Endo K, Kito N, Yanagawa N, Yu Y, Li J, Iwai N (2013) Renoprotective mechanisms of pirfenidone in hypertension-induced renal injury: through anti-fibrotic and anti-oxidative stress pathways. Biomed Res 34(6):309–319. 10.2220/biomedres.34.309 24389407 10.2220/biomedres.34.309

[CR9] Krafft E, Lybaert P, Roels E, Laurila H, Rajamäki Mm, Farnir F, Myllärniemi M, Day Mj, Mc Entee K, Clercx C (2014) Transforming growth factor beta 1 activation, storage, and signaling pathways in idiopathic pulmonary fibrosis in dogs. J Vet Intern Med 28(6):1666–1675. 10.1111/jvim.12432 25331544 10.1111/jvim.12432 PMC4895628

[CR10] Lan HY, Chung AC-K (2012) TGF-β/Smad signaling in kidney disease. Semin Nephrol 32(3):236–243. 10.1016/j.semnephrol.2012.04.002 22835454 10.1016/j.semnephrol.2012.04.002

[CR11] Lee KW, Everett TH, Rahmutula D, Guerra JM, Wilson E, Ding C, Olgin JE (2006) Pirfenidone prevents the development of a vulnerable substrate for atrial fibrillation in a canine model of heart failure. Circulation 114(16):1703–1712. 10.1161/CIRCULATIONAHA.106.624320 17030685 10.1161/CIRCULATIONAHA.106.624320 PMC2129103

[CR12] Leh S, Vaagnes Ø, Margolin SB, Iversen BM, Forslund T (2005) Pirfenidone and candesartan ameliorate morphological damage in mild chronic anti-GBM nephritis in rats. Nephrol Dialysis Transplantation 20(1):71–82. 10.1093/ndt/gfh562 10.1093/ndt/gfh562 15561744

[CR13] Li Z, Liu X, Wang B, Nie Y, Wen J, Wang Q, Gu C (2017) Pirfenidone suppresses MAPK signalling pathway to reverse epithelial-mesenchymal transition and renal fibrosis. Nephrology 22(8):589–597. 10.1111/nep.12831 27245114 10.1111/nep.12831

[CR14] Martins VL, Caley M, O’Toole EA (2013) Matrix metalloproteinases and epidermal wound repair. Cell Tissue Res 351(2):255–268. 10.1007/s00441-012-1410-z 22526628 10.1007/s00441-012-1410-z

[CR15] Meng X, Nikolic-Paterson DJ, Lan HY (2016) TGF-β: the master regulator of fibrosis. Nat Rev Nephrol 12(6). 10.1038/nrneph.2016.48. Article 6 10.1038/nrneph.2016.48 27108839

[CR16] Minkus G, Reusch C, Hörauf A, Breuer W, Darbès J, Kraft W, Hermanns W (1994) Evaluation of renal biopsies in cats and dogs—histopathology in comparison with clinical data. J Small Anim Pract 35(9):465–472. 10.1111/j.1748-5827.1994.tb03952.x

[CR17] O’Neill DG, Elliott J, Church DB, McGreevy PD, Thomson PC, Brodbelt DC (2013) Chronic kidney disease in dogs in UK veterinary practices: prevalence, risk factors, and survival. J Vet Intern Med 27(4):814–821. 10.1111/jvim.12090 23647231 10.1111/jvim.12090

[CR18] Qiu ZZ, He JM, Zhang HX, Yu ZH, Zhang ZW, Zhou H (2019) Renoprotective effects of pirfenidone on chronic renal allograft dysfunction by reducing renal interstitial fibrosis in a rat model. Life Sci 233116666. 10.1016/j.lfs.2019.116666 10.1016/j.lfs.2019.116666 31325427

[CR19] RamachandraRao SP, Zhu Y, Ravasi T, McGowan TA, Toh I, Dunn SR, Okada S, Shaw MA, Sharma K (2009) Pirfenidone is Renoprotective in Diabetic kidney disease. J Am Soc Nephrol 20(8):1765. 10.1681/ASN.2008090931 19578007 10.1681/ASN.2008090931 PMC2723978

[CR20] Sharma K, Ix JH, Mathew AV, Cho M, Pflueger A, Dunn SR, Francos B, Sharma S, Falkner B, McGowan TA, Donohue M, RamachandraRao S, Xu R, Fervenza FC, Kopp JB (2011) Pirfenidone for diabetic nephropathy. J Am Soc Nephrol 22(6):1144–1151. 10.1681/ASN.2010101049 21511828 10.1681/ASN.2010101049 PMC3103734

[CR21] Shihab FS, Bennett WM, Yi H, Andoh TF (2002) Pirfenidone Treatment decreases transforming growth Factor-β1 and matrix proteins and ameliorates fibrosis in chronic cyclosporine nephrotoxicity. Am J Transplant 2(2):111–119. 10.1034/j.1600-6143.2002.020201.x 12099512 10.1034/j.1600-6143.2002.020201.x

[CR22] Shimizu T, Kuroda T, Hata S, Fukagawa M, Margolin SB, Kurokawa K (1998) Pirfenidone improves renal function and fibrosis in the post-obstructed kidney. Kidney Int 54(1):99–109. 10.1046/j.1523-1755.1998.00962.x 9648068 10.1046/j.1523-1755.1998.00962.x

[CR23] Spee B, Arends B, Van Den Ingh TSGAM, Brinkhof B, Nederbragt H, Ijzer J, Roskams T, Penning LC, Rothuizen J (2006) Transforming growth factor β-1 signalling in canine hepatic diseases: New models for human fibrotic liver pathologies. Liver Int 26(6):716–725. 10.1111/j.1478-3231.2006.01277.x 16842329 10.1111/j.1478-3231.2006.01277.x

[CR24] Takakura K, Tahara A, Sanagi M, Itoh H, Tomura Y (2012) Antifibrotic effects of pirfenidone in rat proximal tubular epithelial cells. Ren Fail 34(10):1309–1316. 10.3109/0886022X.2012.718955 23002925 10.3109/0886022X.2012.718955

[CR25] Takakuta K, Fujimori A, Chikanishi T, Tanokura A, Iwatsuki Y, Yamamoto M, Nakajima H, Okada M, Itoh H (2010) Renoprotective properties of pirfenidone in subtotally nephrectomized rats. Eur J Pharmacol 629(1–3):118–124. 10.1016/j.ejphar.2009.12.011 20006961 10.1016/j.ejphar.2009.12.011

[CR26] Tang Q, Markby GR, MacNair AJ, Tang K, Tkacz M, Parys M, Phadwal K, MacRae VE, Corcoran BM (2023) TGF-β-induced PI3K/AKT/mTOR pathway controls myofibroblast differentiation and secretory phenotype of valvular interstitial cells through the modulation of cellular senescence in a naturally occurring in vitro canine model of myxomatous mitral valve disease. Cell Prolif 56(6):e13435. 10.1111/cpr.13435 36869852 10.1111/cpr.13435 PMC10280140

[CR28] US Food and Drug Administration (2017) ESBRIET^®^ (pirfenidone) capsules and film-coated tablets, for oral use. U.S. food and drug administration. https://www.accessdata.fda.gov/drugsatfda_docs/label/2017/208780s000lbl.pdf. Accessed 3 Jan 2024

[CR27] US Food and Drug Administration (2014) 022535Orig1s000. U.S. food and drug administration. https://www.accessdata.fda.gov/drugsatfda_docs/nda/2014/022535Orig1s000ClinPharmR.pdf. Accessed 4 Jan 2024

[CR30] Yhee J-Y, Yu C-H, Kim J-H, Sur J-H (2008) Effects of T lymphocytes, interleukin-1, and interleukin-6 on renal fibrosis in canine end-stage renal disease. J Vet Diagn Investig 20(5):585–592. 10.1177/104063870802000508 18776090 10.1177/104063870802000508

[CR29] Yhee J-Y, Yu C-H, Kim J-H, Im K-S, Chon S-K, Sur J-H (2010) Histopathological retrospective study of canine renal disease in Korea, 2003 ~ 2008. J Vet Sci 11(4):277. 10.4142/jvs.2010.11.4.277 21113095 10.4142/jvs.2010.11.4.277 PMC2998737

