# Supplementary figures and images for: Pirfenidone inhibits TGF-β1-induced fibrosis via downregulation of Smad and ERK pathway in MDCK cells
Source: Vet Res Commun. 2024 Aug 12;48(5):3167–76. doi: 10.1007/s11259-024-10493-y (PMC11442594; doi:10.1007/s11259-024-10493-y)

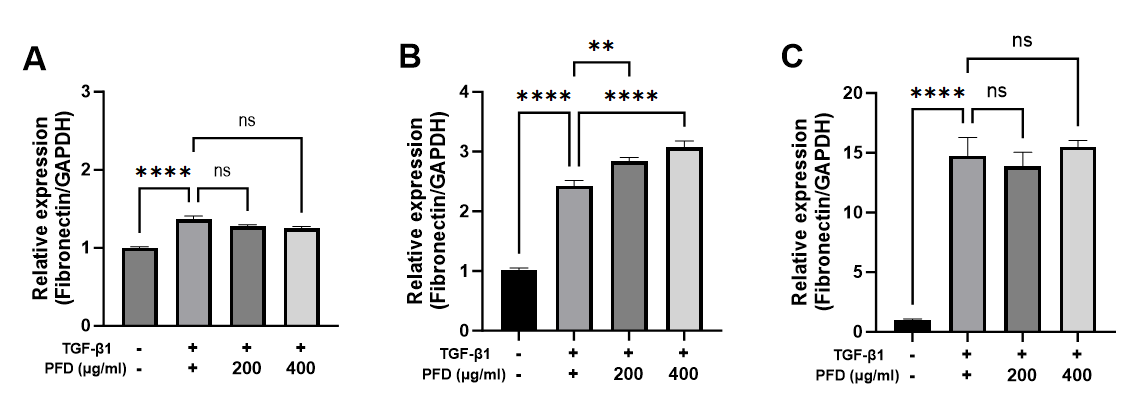

Supplement: Supplementary file 2 — Fig. S1 [file 11259_2024_10493_MOESM2_ESM.png]
